# Some Points of Preventive Treatment in the Diseases of Women

**Published:** 1899-06-03

**Authors:** Arthur E. Giles

**Affiliations:** Assistant Surgeon Chelsea Hospital for Women


### Hospital Clinics and Medical Progress.

### SOME POINTS OF PREVENTIVE TREATMENT IN THE DISEASES OF WOMEN.

By ARTHUR E. GILES, M.D., B.Sc., M.R.C.P.Lond., F.R.C.S.Ed., Assistant Surgeon Chelsea Hospital for Women.

IN THE HOSPITAL for April 10th, 1897, I dwelt on some directions in which preventive treatment might be applied to the question of diseases of women, viz., with respect to dysmenorrhœa, to gonorrhœa, and to miscarriage.

Some further aspects of preventive treatment well deserve consideration, and one of the most important is the prevention, by proper attention at the time of labour, of diseases resulting from child-bearing.

**Diseases Resulting from Child-bearing.**—Semmelweiss, when resident in the maternity department of the Vienna Hospital, was driven to investigate the cause of puerperal fever by the repeated and fatal sound of the bell which summoned the priest to perform the last rites for the dying. Its ominous tolling weighed on his heart and brain; and it is now well-known history how his efforts, born of his suffering pity, led to the salvation of thousands from the most tragic death that can befall a woman.

Puerperal fever has been fought and subdued; but its power of destruction is not gone, and annually many lives are still sacrificed through a preventable disease. Carelessness is still in part to blame, and ignorance more largely. It is still not superfluous to urge medical men to leave no precaution untried when attending women in labour. And it is far from superfluous to urge upon legislators the necessity of controlling those who, with no training whatever, may label themselves "midwife," and may in gross ignorance attend poor mothers at the time when they most need skilful and careful attention. In this matter I do not propose to enter into debated questions of medical politics; whether midwives be registered, or whether an order of midwifery nurses be created, is a very small matter compared to the main point, which is that means be taken to ensure that poor women in their confinement be not attended by the ignorant and careless.

This is all I need say now on this point; for there are other diseases and dangers associated with child-bearing which claim our attention. To those who have much to do with the treatment of the diseases of women this statement must have come with something of the painful iteration of Semmelweiss' passing-bell: "I have never been right since my confinement." It is with these words ringing in my ears that I am urged to consider some of the conditions for which child-bearing is responsible, and the means to be adopted to prevent them.

It will best answer my purpose to begin with a list of these conditions. They are: Injuries at the time of labour, such as tearing of cervix, vagina, or perineum; subinvolution; chronic endometritis and erosion of the cervix; prolapse of the vaginal walls; prolapse and procidentia of the uterus; pelvic cellulitis; and vesico-vaginal and recto-vaginal fistula.

These conditions derive their importance not so much from their seriousness as from their frequency and their long duration. The out-patient departments of hospitals are always well supplied with such cases, and they also occur largely in private practice. When not treated the patient is often invalided for years, and even under treatment the disease may be very obstinate.

Several groups of sequences are found. Thus, firstly, the cervix may be much torn; this leads to eversion of the cervical mucous membrane, to erosion, and to chronic endometritis. Secondly, the vagina may be torn, and the tear in the cervix may extend into the pelvic cellular tissue, leading or predisposing to pelvic cellulitis. Thirdly, the perineum may be torn, predisposing to prolapse of the vaginal walls and uterus, especially in those who have borne many children, and favouring endometritis. Subinvolution forms a separate group, and is brought about by too early resumption of duties or by want of care in the treatment during the period of lying-in.

What can be done in the direction of prevention? The first and most important thing is that women should be attended in their confinement by competent accoucheurs or midwives. This opens up two great questions of medical policy, namely, the more thorough training of medical students in midwifery, and an alteration in the present order of things as far as midwives are concerned. I need not enlarge further on what I have already said on the latter point; but a plea may be suitably put in for the better training of students. Many a student attends the six, twelve, or twenty cases of labour required by examination boards without having even attended a course of lectures on the subject; and even if he has already received theoretical instruction, he has often to attend the confinements alone, or with no more supervision than is afforded by a midwife, which often means with no supervision at all. How can he learn properly under such circumstances? The result, when he starts practice, may be readily foretold. Of course, he learns by experience; but why should the price of that experience be paid by his early patients, too often in the form of chronic invalidism? We take it that this is a subject for the consideration of the General Medical Council. The Council has done much. Will it not go on to do more in a matter of such importance?

There are, of course, even graver issues involved in the question of proper attendance on women in labour, issues of life and death arising out of the complications of labour. These I cannot now go into, as they do not come within the scope of my subject, and so I pass on to the second suggestion as to the prevention of bad after-effects of labour. It is, briefly, that in childbirth as much as possible should be left to nature. Unnecessarily frequent examinations should be studiously avoided. During the first stage, with the membranes unruptured, with a normal presentation and an uncontracted pelvis, no interference should be made as long as the patient's general condition is satisfactory. It may be said that with so many provisos the admonition is surely superfluous; but I think not. On the contrary, I believe that a busy man too often yields to the importunity of the patient, and to the temptation to hurry on matters; the os is not allowed to dilate naturally to its full extent, but the membranes are ruptured, the forceps applied, and delivery completed. The result is almost certain laceration of the cervix, the laceration being extensive, and perhaps involving the vaginal vault; and thus is started one of the unwelcome sequences above mentioned. The likelihood of rupture of the perineum is also increased by this prematurely rapid delivery, for the lower parts of the vagina have no time to stretch and adapt themselves to the coming head. When a perineal tear occurs in this way it is very likely to extend into the rectum. Thus a whole train of troublesome symptoms, followed by the constant wearing of a pessary, or by an operation later on, is brought about by the desire to shorten the labour by an hour. From the patient's point of view it would often be better to spend an extra half-day over the confinement. Similarly in the second stage, reasonable time should be allowed for the natural dilatation of the parts, and the forceps only applied when there is a definite indication; the mere shortening of the second stage, independently of other considerations, ought not to be regarded as an indication for the use of the forceps.

Finally, in the third stage, when the patient is comfortable, when there is no hæmorrhage, when the pulse is good, and the uterus reasonably contracted, proper time should be allowed for the natural expulsion of the placenta. I am firmly convinced that, from the point of view of the subsequent health of the patient, it is of the utmost importance that from first to last as much as possible should be left to the natural forces of delivery.

